# Chronic disease self-management education courses: utilization by low-income, middle-aged participants

**DOI:** 10.1186/s12939-017-0604-0

**Published:** 2017-06-27

**Authors:** Lindsey N. Horrell, Shawn M. Kneipp, SangNam Ahn, Samuel D. Towne, Chivon A. Mingo, Marcia G. Ory, Matthew Lee Smith

**Affiliations:** 1 School of Nursing, The University of North Carolina at Chapel Hill, Chapel Hill, North Carolina USA; 2 School of Public Health, The University of Memphis, Memphis, Tennessee USA; 3 School of Public Health, Texas A&M University, College Station, Texas USA; 4 Gerontology Institute, College of Arts and Sciences, Georgia State University, Atlanta, Georgia 30302 USA; 5 College of Public Health, The University of Georgia, Athens, GA USA

**Keywords:** Chronic disease, Self-management, Low-income, Health disparities, Chronic Disease Self-management Education Courses: Utilization by Low-income, Middle-aged Participants

## Abstract

**Background:**

Individuals living in lower-income areas face an increased prevalence of chronic disease and, oftentimes, greater barriers to optimal self-management. Disparities in disease management are seen across the lifespan, but are particularly notable among middle-aged adults. Although evidence-based Chronic Disease Self-management Education courses are available to enhance self-management among members of this at-risk population, little information is available to determine the extent to which these courses are reaching those at greatest risk. The purpose of this study is to compare the extent to which middle-aged adults from lower- and higher-income areas have engaged in CDSME courses, and to identify the sociodemographic characteristics of lower-income, middle aged participants.

**Methods:**

The results of this study were produced through analysis of secondary data collected during the *Communities Putting Prevention to Work: Chronic Disease Self-Management Program* initiative. During this initiative, data was collected from 100,000 CDSME participants across 45 states within the United States, the District of Columbia, and Puerto Rico.

**Results:**

Of the entire sample included in this analysis (19,365 participants), 55 people lived in the most impoverished counties. While these 55 participants represented just 0.3% of the total study sample, researchers found this group completed courses more frequently than participants from less impoverished counties once enrolled.

**Conclusion:**

These results signal a need to enhance participation of middle-aged adults from lower-income areas in CDSME courses. The results also provide evidence that can be used to inform future program delivery choices, including decisions regarding recruitment materials, program leaders, and program delivery sites, to better engage this population.

## Background

Individuals who reside in impoverished areas are at higher risk of facing a number of chronic health conditions (e.g. cardiovascular disease, diabetes, cancer, etc.), a disparity likely linked to the robust association between individual income and income-based community segregation. [1–3] In addition, the higher levels of chronic stress associated with having lower-incomes can lead to an earlier-onset and accelerated progression of chronic conditions when compared to adults who live in more socioeconomically advantaged neighborhoods. [3–6] Despite the higher prevalence of chronic disease among lower-income adults, they often have fewer opportunities to engage in health promotion and/or disease management programs in the places where they live and work. [4–6] This reality, along with the limited access to healthy food options and/or safe areas for physical activity that many living in impoverished areas face, are some of the more salient of the many barriers to optimal management of chronic disease faced by lower-income adults. [7–9]

While healthcare disparities across region and socioeconomic classes are observed throughout the lifespan [10–14], the gap in chronic disease burden widens in middle adulthood. [15, 16] This stage of life is often accompanied by elevated stress related to work, social and familial demands that can trigger chronic disease onset, exacerbate the effects of disease-related morbidity, and distract from disease self-management. [17–20] These realities often leave middle-aged adults living in lower-income areas at increased risk of suffering worse health outcomes related to chronic disease than those living in higher-income areas.

Evidence-based community interventions are available that can help middle-aged adults better manage their chronic conditions; however, such programs are often not utilized by individuals from lower-income areas as often as participants from higher-income areas. [21] Chronic Disease Self-management Education (CDSME) courses, for example, have been linked to a number of improved health outcomes among middle-aged participants. [22] The core of these courses, the Stanford Chronic Disease Self-Management Program (CDSMP), is a peer-led workshop offered in six, 2.5 h weekly sessions and incorporates a number of lessons such as medication adherence, communication skills to converse with medical professionals, and nutrition tips to help cope with chronic conditions, among others. [23] While CDSME courses have proven to be effective in eliciting positive health outcomes such as increased self-efficacy, decreased fatigue, and decreased healthcare utilization, [24] little is known about the extent to which middle-aged participants from lower income areas have engaged in CDSME courses.

### Purpose

The aim of this research is to compare enrollment and completion rates of middle-aged CDSME participants living in lower-and higher-income areas and to specifically describe key sociodemographic characteristics of those residing in lower-income areas. The study is guided by the World Health Organization’s Framework on the Social Determinants of Health [25] and broad ecological theory [26]. Because poverty is a main social determinant of health, it was used to identify areas likely to have less access to resources or poorer health-related outcomes. With rates of chronic disease climbing among middle-aged adults, ensuring that evidence-based self-management programs are extended to those living in lower-income areas is critical in mitigating the disproportionate chronic disease burden facing this population. [27] Thus, the purpose of this study is:

Aim 1 To identify how enrollment and CDSME course completion compares between middle-aged participants living in lower-and higher-income areas.

Aim 2 To describe sociodemographic characteristics of middle-aged adults from lower-income areas who participate in the CDSME.

Better understanding patterns of CDSME participation will inform future marketing efforts to promote enrollment among those living in lower-income areas.

## Methods

### Data source

The data used in this secondary analysis were collected during a national roll-out of CDSME programs through the American Recovery and Reinvestment Act of 2009 (ARRA) *Communities Putting Prevention to Work: Chronic Disease Self-Management Program* initiative. [23, 28] During this rollout, data describing the delivery site, program attendance, and participants were collected from the first 100,000 participants. [23, 28] The programs were offered in several languages across 45 states within the United States, the District of Columbia and Puerto Rico and include both generic and condition-specific (e.g., diabetes, HIV, pain) self-management courses. [23, 28]

### Measures

The primary independent variables for this study included course attendance (i.e., number of sessions attended of six offered sessions), course site (e.g., senior center, healthcare organization, residential facility, faith-based organization), and participant sociodemographic information (i.e., age, sex, race/ethnicity, chronic conditions). These variables were selected based on previous studies documenting their relationship to CDSME enrollment and participation. [23, 29, 30] Data were collected at the CDSME delivery site and later entered into an online national database for tracking and analysis. [23, 28]

The dependent variable for this study was the proportion of households in the participants’ county of residence that lived below 125% of the poverty line. Administrators geocoded the dataset to add the median household income in the participant’s county of residence. Participants were then categorized by the percent of individuals living in their county of residence who lived below 125% of the poverty line defined by the U.S. Department of Health and Human Services. [31–33] The 125% poverty threshold was chosen as a proxy for the 138% poverty threshold to qualify for federal assistance programs such as Medicaid. [31–33] For this study, ‘area’ was defined at the county level, where CDSME participation data were available for analysis. From this point forward, counties with over half of the population under the 125% poverty threshold are referred to as the “most impoverished” counties, and counties with less than one-quarter of the population living below the 125% poverty threshold are referred to as the “least impoverished.” As such, we use these extreme ends of the county-level income spectrum to make comparisons, where counties with a higher proportion of middle-income households may be less represented.

### Analyses

Analyses were performed using SPSS (Version 22). [34] Only participants between the ages of 50 and 64 years with reported county-level data were included in analyses. All cases not meeting these inclusion criteria were omitted. “Area impoverishment” categories were constructed by dividing the study sample into quartiles based on the percent of households at or below the 125% poverty threshold within each CDSME participant’s county of residence. Frequencies and descriptive statistics were calculated for all variables of interest, which were then compared across quartiles. To address the first aim, Chi-square tests were used to assess the distribution differences for categorical variables, and one-way ANOVA were used to assess mean differences by area impoverishment for continuous and count variables (Table 1). To address the second aim, an ordinal regression model was fitted to compare sociodemographic and participation factors across quartiles, with higher quartiles indicating more poverty (Table 2). Finally, because the lowest quartile (Quartile 4) included a wider range of counties across the area impoverishment continuum, which may mask true similarities and differences between those living in the most impoverished counties and those living in lesser-impoverished areas, this quartile was further broken down into three sub-groups based on percentage of the county living under the 125% poverty threshold (Table 3). This sub-analysis was conducted to further examine the differences between participants living in the most impoverished areas (over 50% of households under the poverty threshold), and those living in less impoverished areas within this quartile. Frequencies and descriptive statistics were again run for all variables of interests, and mean and distribution differences were assessed using the same methods described for Table 1.

**Table 1:** Sample characteristics by county-level poverty status (in quartiles)

		Percent of the population under 125% poverty					
	Total (*n* = 19,365)	Quartile 1 (*n* = 4,760)	Quartile 2 (*n* = 5,029)	Quartile 3 (*n* = 4,924)	Quartile 4 (*n* = 4,652)	*χ* ^2^ or f	*P*
Percent of county under 125% poverty							
Lowest percent	3.51%	3.51%	15.29%	19.51%	22.63%		
Highest percent	69.12%	15.27%	19.50%	22.62%	69.12%		
Age	58.49 (±4.25)	58.43 (±4.24)	58.56 (±4.22)	58.63 (±4.24)	58.34 (±4.29)	4.40	0.004
Sex						8.06	0.045
Male	23.0%	23.5%	24.0%	21.7%	22.9%		
Female	77.0%	76.5%	76.0%	78.3%	77.1%		
Ethnicity						137.10	<0.001
Non-hispanic	88.3%	92.3%	88.7%	87.9%	84.1%		
Hispanic	11.7%	7.7%	11.3%	12.1%	15.9%		
Race						1094.37	<0.001
White	62.5%	67.4%	68.4%	59.6%	54.3%		
African american	23.7%	14.0%	18.7%	27.0%	35.3%		
Asian/pacific islander	4.4%	9.4%	4.6%	2.7%	0.8%		
American indian/native american	2.0%	1.8%	1.7%	1.9%	2.5%		
Other/mixed race	7.5%	7.4%	6.6%	8.8%	7.1%		
Number of chronic conditions (0 to 10)	2.57 (±1.68)	2.55 (±1.65)	2.56 (±1.70)	2.67 (±1.70)	2.48 (±1.66)	9.57	<0.001
No conditions	8.2%	7.9%	8.7%	8.1%	8.2%	51.94	<0.001
1 Condition	21.7%	22.6%	21.8%	19.5%	23.2%		
2 Conditions	23.4%	22.4%	23.0%	22.7%	25.4%		
3+ Conditions	46.7%	47.2%	46.5%	49.6%	43.2%		
Delivery site type						635.75	<0.001
Senior center/AAA	24.0%	19.6%	26.3%	22.4%	27.5%		
Healthcare organizations	26.3%	30.7%	26.9%	25.9%	21.5%		
Residential facility	12.0%	13.1%	13.5%	10.0%	11.2%		
Community/multi-purpose facility	11.5%	11.7%	10.8%	15.3%	8.3%		
Faith-based organization	9.8%	10.7%	8.2%	10.1%	10.5%		
Educational institution	2.7%	2.2%	1.4%	3.6%	3.5%		
County health department	1.8%	2.1%	2.1%	1.8%	1.0%		
Tribal organization	0.3%	0.1%	0.7%	0.3%	0.2%		
Other delivery site	10.8%	8.2%	9.4%	10.0%	15.9%		
Workplace	0.9%	1.6%	0.7%	0.7%	0.5%		
Median household income level (in $10,000 increments)	5.02 (±1.24)	6.50 (±1.20)	5.06 (±0.62)	4.71 (±0.59)	3.80 (±0.56)	9652.62	<0.001
Percent of population with less than high school education	3.59% (±7.80)	1.85% (±3.32)	2.53% (±3.81)	6.91% (±13.08)	3.03% (±5.48)	441.31	<0.001
Number of sessions attended (of 6 offered sessions)	4.46 (±1.66)	4.46 (±1.65)	4.44 (±1.67)	4.40 (±1.69)	4.52 (±1.60)	4.24	0.005
Non-successful completion (attending less than 4 sessions)	23.5%	23.4%	23.9%	25.1%	21.6%	16.51	0.001
Successful completion (attending 4+ sessions)	76.5%	76.6%	76.1%	74.9%	78.4%

**Table 2:** Ordinal regression for 125% poverty quartiles

	Beta	SE		*P*	95% CI	
			Wald		Lower	Upper
Age	0.02	0.00	13.94	<0.001	0.01	0.02
Female	0.03	0.04	0.52	0.471	−0.05	0.11
Male	0.00	--	--	--	--	--
Hispanic	0.88	0.06	208.75	<0.001	0.76	1.00
Non-hispanic	0.00	--	--	--	--	--
Other/mixed race	0.60	0.07	66.98	<0.001	0.46	0.74
American indian/native american	0.27	0.13	4.56	0.035	0.02	0.53
Asian/pacific islander	0.93	0.10	87.61	<0.001	0.73	1.12
African american	1.22	0.04	770.18	<0.001	1.13	1.30
White	0.00	--	--	--	--	--
Number of chronic conditions	−0.02	0.01	2.66	0.103	−0.04	0.00
Median household income for county	−3.34	0.04	8621.65	<0.001	−3.41	−3.27
Percent of county without high school education	0.11	0.00	1082.74	<0.001	0.10	0.12
Workplace	−0.55	0.18	8.93	0.003	−0.91	−0.19
Other delivery site	0.40	0.06	39.87	<0.001	0.27	0.52
Tribal organization	0.23	0.28	0.70	0.403	−0.31	0.78
County health department	−0.40	0.12	10.28	0.001	−0.64	−0.15
Educational institution	0.45	0.11	18.14	<0.001	0.24	0.66
Faith-based organization	−0.02	0.07	0.90	0.762	−0.15	0.11
Community/multi-purpose facility	0.20	0.06	10.63	0.001	0.08	0.31
Residential facility	0.15	0.06	6.59	0.010	0.04	0.27
Healthcare organizations	0.05	0.05	0.90	0.342	−0.05	0.14
Senior center/AAA	0.00	--	--	--	--	--
Successful workshop completion	−0.13	0.04	10.08	0.002	−0.21	−0.05
Non-successful workshop completion	0.00	--	--	--	--	--
Nagelkerke R^2^ = 0.755

**Table 3:** Sample characteristics by county-level poverty status (fourth quartile only)

		Percent of the population under 125% poverty				
	Total (*n* = 4,652)	0% to 24.9% (*n* = 1,477)	25.0% to 49.9% (*n* = 3,120)	50.0% to 74.9% (*n* = 55)	*χ* ^2^ or f	P
Percent of county under 125% poverty						
Lowest percent	22.63%	22.63%	25.00%	50.16%		
Highest percent	69.12%	24.96%	49.43%	69.12%		
Age	58.34 (±4.29)	58.42 (±4.23)	58.27 (±4.32)	60.07 (±3.98)	5.20	0.006
Sex					4.87	0.088
Male	22.9%	22.5%	23.3%	10.9%		
Female	77.1%	77.5%	76.7%	89.1%		
Ethnicity					402.39	<0.001
Non-hispanic	84.1%	95.9%	79.7%	10.9%		
Hispanic	15.9%	4.1%	20.3%	89.1%		
Race					136.35	<0.001
White	54.3%	64.8%	49.3%	54.5%		
African american	35.3%	27.8%	39.2%	18.2%		
Asian/pacific islander	0.8%	0.5%	1.0%	0.0%		
American indian/native american	2.5%	1.7%	2.9%	0.0%		
Other/mixed race	7.1%	5.1%	7.6%	27.3%		
Number of chronic conditions (0 to 10)	2.48 (±1.66)	2.63 (±1.65)	2.42 (±1.66)	2.15 (±1.50)	9.57	<0.001
No Conditions	8.2%	6.0%	9.2%	9.1%	27.72	<0.001
1 Condition	23.2%	21.4%	23.9%	32.7%		
2 Conditions	25.4%	25.0%	25.6%	21.8%		
3+ Conditions	43.2%	47.6%	41.3%	36.4%		
Delivery site type					126.71	<0.001
Senior center/AAA	27.5%	32.7%	25.3%	9.1%		
Healthcare organizations	21.5%	22.7%	21.1%	7.3%		
Residential facility	11.2%	9.3%	12.3%	0.0%		
Community/multi-purpose facility	8.3%	8.5%	7.9%	21.8%		
Faith-based organization	10.5%	9.1%	11.2%	9.1%		
Educational institution	3.5%	3.8%	3.4%	3.6%		
County health department	1.0%	1.0%	1.1%	0.0%		
Tribal organization	0.2%	0.2%	0.2%	0.0%		
Other delivery site	0.5%	0.7%	0.4%	0.0%		
Workplace	0.5%	0.7%	0.4%	0.0%		
Median household income level (in $10,000 increments)	3.80 (±0.56)	4.12 (±0.34)	3.68 (±0.51)	1.73 (±0.35)	1022.91	<0.001
Percent of population with less than high school education	3.03% (±5.48)	2.63 (±5.54)	3.27 (±5.47)	0.38 (±0.35)	13.45	<0.001
Number of sessions attended (of 6 offered sessions)	4.52 (±1.60)	4.49 (1.61±)	4.52 (±1.60)	5.18 (±1.35)	4.95	0.007
Non-successful completion (attending less than 4 sessions)	21.6%	23.1%	21.1%	12.7%	5.03	0.081
Successful completion (attending 4+ sessions)	78.4%	76.9%	78.9%	87.3%		

## Results

Of the 100,000 participants included in this national data set, 19,365 (19.4%) were aged 50 to 64 years and eligible for inclusion in this secondary analysis, which is similar to the ages of participants found in other studies of CDSME courses [22]. Looking at Table 1, it is clear the majority of these participants resided in the least impoverished counties included in this analysis (i.e., where less than 25% of the population lived under the 125% poverty threshold), with all participants in Quartiles 1, 2 and 3 falling into this category. While little variance was shown in age and gender of participants across quartiles in Table 1, more impoverished counties did include more participants who identified as Hispanic, African American, and American Indian/Native American, and fewer who identified as non-Hispanic, White and Asian/Pacific Islander. The majority of participants across quartiles experienced 2–3 chronic conditions at the point of data collection.

Participants residing in the least impoverished areas (Table 1, Quartile 1) were more likely (*p* <0.001) to attend courses at healthcare organizations than participants in more impoverished areas. Conversely, participants in more impoverished areas (Table 1, Quartile 4), were more likely to attend sessions in “other” delivery sites (personal residences, casinos, fire departments, malls, etc.) than those residing in less impoverished areas (*p* <0.001). These findings are consistent with the ordinal regression results displayed in Table 2. Looing again at Tables 1 and 2, a step-wise decrease in median individual-level household income was seen in participants from Quartiles 1–4 (*p* <0.001). While no trend in successful program completion was identified, CDSME program completion was slightly higher (at 78.4%) among participants living in more impoverished counties represented in Quartile 4 when compared to participants in other quartiles (see Table 1).

Table 2 displays findings from the ordinal regression model to assess the association between sociodemographic and program participation factors and levels of area impoverishment. Across levels, a higher proportion of participants who identified as a racial/ethnic minority group member resided in more impoverished counties (*p* < .001). Additionally, those living in areas with higher percentages of residents without a high school education also resided in more impoverished counties (*p* < 0.001). Participants who resided in more impoverished counties were also significantly more likely to attend workshops at residential facilities (*p* = 0.010), community/multi-purpose facilities (*p* = 0.001), educational institutions (*p* < 0.001) and “other” delivery sites compared to participants in less impoverished areas, who were more likely to attend CDSMP courses in county health departments (*p* = 0.001) and workplaces (*p* < 0.001).

Looking again at Table 1, the representation of CDSME participants across the different levels of area impoverishment revealed more variability in Quartile 4 than was represented in other quartiles. Those in Quartile 1, for example, resided in counties with 3.51%–15.27% (range = 11.76) of the population living below the 125% poverty threshold; in Quartile 2 from 15.29%–19.50% (range = 4.21); in Quartile 3 from 19.51%–22.62% (range = 3.11); and in Quartile 4 from 22.63–69.12% (range = 46.49). Because the range in the fourth quartile was much larger than that of the first three, we conducted a sub-analysis of Quartile 4 in which the participants were sub-divided into three categories (in increments of 25%) based on the proportion of households residing in poverty to determine if any significant differences existed among participants residing in the most severely impoverished areas. It should be noted that no participants resided in counties with 75% or more households under 125% poverty. Results of this sub-analysis are displayed in Table 3.

According to this sub-analysis of Quartile 4, an additional 1,477 participants from Quartile 4 lived in counties where less than 25% of households lived below the poverty threshold. When combined with the 14,713 participants included in Quartiles 1, 2 and 3, the results of this sub-analysis signal that a total of 83.6% of the 19,365 participants included in this study resided in the least impoverished counties. Furthermore, only 55 participants lived in the most impoverished counties (where over 50% of households lived under the poverty threshold), representing only 0.3% of the total study sample. This group residing in the most impoverished counties included more female (89.1%) and Hispanic (89.1%) participants than others within the sub-analysis, as well as more participants who self-identified as being a mixed or ‘other’ race (27.3%). Participants from the most impoverished counties also attended programs held at community/multi-purpose facilities most-frequently (21.8%), whereas participants from lesser-impoverished counties within the fourth quartile attended courses at senior centers, healthcare organizations and residential facilities more frequently. Participants living in the most impoverished counties earned smaller median household incomes, but these counties did not have higher percentages of adults without a high school education.

## Discussion

The results of this study reveal that middle-aged CDSME course participants from the most impoverished counties, who were most likely to be exposed to socioeconomic determinants that could lead to poorer chronic disease management, may be more likely to complete CDSME courses once enrolled than those from counties with lower poverty rates. Arguably of most interest in the sub-analysis of Quartile 4 is the fact that, although not statistically significant (likely due to small cell sizes), there was a trend in participants residing in the most impoverished counties being more likely to complete the CDSME program (87.3%) when compared to other sub-groups (76.9% and 78.9%). With this said, less than 1% of participants attending CDSME courses reside in the most impoverished areas using the definition chosen in the current study. Thus, despite the known detrimental effects that living in a highly impoverished area have on health behaviors and outcomes, and despite the high completion rates demonstrated by participants from the most impoverished areas, CDSME courses fail to engage high percentages of participants living in highly impoverished settings.

Only one county in the U.S. currently has greater than a 50% poverty rate, which aligns with the county-level income distribution observed in this study. [35] However, individuals living in areas of higher poverty, where many may have already limited access to health resources, may benefit most from CDSME courses. Thus, future efforts should be made to better engage this population. It may be concluded from this analysis; however, that changes do not need to be made to the content of CDSME courses to further engage this group, as the majority of participants residing in counties with the highest poverty rates completed the CDSME course once enrolled (87.3%). Rather, efforts should focus on CDSME marketing strategies that are appealing and acceptable to participants from lower-income areas to increase enrollment of this at-risk population. Moreover, the accessibility of the CDSME program is also imperative. Therefore, results from this study not only provide support for consideration of the appeal and acceptability of the marketing strategies, but they also highlight the need to strongly consider delivering the program at locations that would enhance the feasibility of access and achieve greater reach among the target population. The results of this analysis provide valuable evidence concerning the characteristics of middle-aged CDSME participants living in communities with more saturated poverty that could be used to guide these marketing efforts.

### Marketing Solutions

To identify marketing strategies that can be used to increase future enrollment of lower-income, middle-aged adults in CDSME programs, one may look at the sociodemographic characteristics of the sample included in this study. A greater percentage of participants identified as Hispanic and/or African American within lower-income counties compared to participants from wealthier regions. Thus, CDSME leaders may need to review recruitment materials to ensure the images and messages displayed in study advertisements appeal to a multi-ethnic audience. Furthermore, officials should ensure recruitment is taking place in areas frequented by individuals of all racial and ethnic backgrounds. Similar health promotion interventions have successfully recruited participants of minority racial and ethnic backgrounds through barbershops, beauty salons, churches, and local fitness centers. [36–40] Future recruitment efforts may target similar, non-traditional venues in addition to brainstorming new, un-tapped venues in an effort to increase enrollment of this underrepresented population. Partnering with organizations that already service target areas may also provide a much-needed avenue to reach these target populations.

In addition to reviewing recruitment strategies and materials, public health officials should strive to diversify CDSME program delivery sites in an attempt to increase access to available courses and enroll participants from lower-income areas. As displayed in Table 2, participants from counties with higher rates of poverty attended courses held at community/multi-purpose facilities, educational institutions, residential facilities and “other” site locations at notably higher rates than their counterparts from more affluent areas. Furthermore, they attended courses held in workplaces and county health departments at lower rates than participants from the least impoverished areas. Residents of more impoverished areas also attended courses held in senior centers and healthcare organizations far less frequently than other participants. Thus, researchers and program managers hoping to engage adults from lower-income areas may need to become creative in choosing delivery sites that appeal and are readily accessible to this population. While some of the sites suggested above for recruitment may also be ideal delivery sites, additional suggestions may include local sport facilities and residential areas easily accessible by public transportation.

Ensuring that participants can identify with those offering CDSME courses may also be crucial to increasing participation of individuals from lower-income areas. According to Albert Bandura’s concept of observational learning, people more readily learn and follow behavior modeled by people with whom they can identify. [41, 42] Thus, hiring course leaders who reflect the characteristics of the target population may more readily draw them to the program. Further research should be conducted to ascertain the demographic and socioeconomic characteristics of current CDSME course leaders, and efforts should be made to ensure that leaders are available who match the target demographic by race, ethnicity, age, and income. Course leaders should also be trained in cross-cultural communication and cultural awareness to create a welcoming environment for participants of all socioeconomic and ethnic backgrounds.

At this point in this discussion, little has been mentioned regarding the sex of participants from both lower- and higher-income areas. Four out of the five leading causes of death among males are chronic diseases, and over half of preventable deaths related to heart disease and stroke occur in males. [43, 44] Despite this fact, females outnumbered males at greater than a 3:1 ratio while participating in one of the leading evidence-based solutions to enhance chronic disease self-management, a rate reflected in other CDSME research as well. [24] Thus, future CDSME recruitment efforts should focus on recruiting males in addition to lower-income populations. For example, in efforts to recruit males to evidence-based programs, one study interviewing key stakeholders found that over 90% of respondents suggested that simply adapting program advertisements to include images of older men may be more likely to attract male participants [45]. Consistent with other suggestions, this may be achieved through altering program marketing strategies, including targeting different recruitment sites, including images of racially and ethnically diverse males and females in program advertisements, developing recruitment messages tailored to the motivations of target populations, hiring more male course leaders, and diversifying program delivery sites to appeal to both gender groups.

### Limitations

There are several study limitations that must be noted. First, individual data collected during the ARRA initiative were limited in an attempt to reduce participant burden and attract more people to the CDSME courses. Thus, information about annual income was not collected from participants, and conclusions cannot be drawn regarding the association between individual income and program participation practices. Participants from lower-income areas did significantly differ from participants living in higher-income areas on several key demographic, socioeconomic, and attendance variables, however, providing clues to program leaders regarding how to tailor future interventions to participants from lower-income areas. Further research should be conducted to investigate the relationship between individual income and program participation as well as what additional community factors predict program participation. Further, given the ecological fallacy, we cannot assume the income of the county was reflective of program participants or the resources they have access to. Similarly, although participants across counties all experienced an average of approximately 2–3 chronic conditions, the impact of these conditions may vary significantly across groups, but we do not have this information to report. Finally, information about other health promotion and disease prevention programs being offered in the same areas as CDSME courses was not gathered during data collection. Thus, it is unclear whether a lack of engagement of participants from the most impoverished counties stemmed from a higher number of competing interventions being offered in these areas. Regardless, CDSME courses have been associated with improved health and healthcare utilization outcomes among adults of all ages [22], and this study reveals high completion rates among middle-aged adults from low-income areas once they are enrolled. Thus, these limitations should be taken under consideration when interpreting the findings of this research, and future research should focus on identifying barriers to recruitment of individuals from lower income areas to CDSME courses and developing effective strategies to overcome such barriers.

## Conclusions

As chronic disease rates continue to rise among middle-aged adults, CDSME courses offer a promising solution to curb the morbidity-related effects of chronic conditions. Despite the improved health outcomes reported among middle-aged CDSME participants, [22] this study reveals that middle-aged adults residing in highly impoverished areas, who are at an increased risk of suffering negative chronic disease outcomes, remain woefully under-represented in CDSME samples. Only 1% of participants included in this analysis resided in the most impoverished counties, and the vast majority of participants resided in counties where less than one-quarter of the residents lived under the 125% poverty threshold. When enrolled, however, participants from counties with the highest poverty rates completed the CDSME more frequently than participants from less-impoverished areas, signaling that barriers to engagement occur before enrollment, and additional efforts should be made to market CDSME programs in a way that targets adults in lower-income areas. Differences in the sociodemographic characteristics of participants from lower- and higher-income areas identified in this study may be used to guide future CDSME marketing strategies. Implementing such targeted marketing techniques could increase engagement of middle-aged adults from lower-income areas and begin curbing geographic and socioeconomic disparities in chronic disease management.

## Abbreviations

CDSME: Chronic Disease Self-management Education
CDSMP: Chronic Disease Self-management Program
CHIP: Children’s Health Insurance Program
